# The Acceptability of Musculoskeletal Community Appointment Days: A Mixed‐Methods Service Evaluation

**DOI:** 10.1002/msc.70152

**Published:** 2025-07-06

**Authors:** Simon Ingram, Jonathon Gill, Isadora Stathers, Samantha Jenkins, Eleanor Neville, Alice Berry

**Affiliations:** ^1^ Physiotherapy Department Somerset NHS Foundation Trust Taunton UK; ^2^ School of Health and Social Wellbeing University of the West of England Bristol UK

**Keywords:** acceptability, CAD, community appointment day, musculoskeletal, personalised care, physiotherapy, self‐management

## Abstract

**Background:**

Musculoskeletal (MSK) conditions represent an increasing challenge within the NHS. Rising demand has driven a national shift towards more community‐based care and supported self‐management. Community Appointment Days (CAD) are community‐based, full‐day events providing access to clinical expertise, community partners and voluntary sector services. The collaborative ethos facilitates a personalised and holistic approach to musculoskeletal health. This evaluation aimed to explore the acceptability of the CAD model.

**Methods:**

Following a CAD in the Southwest of England, patient experience was collected using the Friends and Family test and CollaboRATE measure for level of shared decision‐making. Interviews were conducted with patients, and a focus group was held with staff. The Theoretical Framework of Acceptability (TFA) informed the interview and focus group guides, and data analysis. Thematic analysis employed a hybrid deductive/inductive approach.

**Results:**

The CAD was attended by 130/160 (81%) patients. Experience measures were completed by 97/130 (75%) patients. The Friends and Family test demonstrated that most patients were ‘extremely likely’ (*n* = 63, 65%) or ‘likely’ (*n* = 32, 33%) to recommend a CAD. The average CollaboRATE score was 10.8/12. One‐to‐one interviews were completed with 13 patients, and a focus group with 10 staff. Four themes were identified: (1) a positive response to the CAD ethos; (2) the importance of effective planning and communication; (3) effective implementation of the CAD; and (4) potential impact and integration with existing musculoskeletal pathways.

**Conclusion:**

The CAD was perceived as an acceptable, personalised, and holistic model of care that supports self‐management and cross‐sector collaboration for supporting MSK health.

## 1 Introduction

Musculoskeletal (MSK) conditions, such as back pain and osteoarthritis, affect over 20 million people in the United Kingdom (Versus Arthritis 2024). With an ageing population and increasing multimorbidity, the impact of MSK health to the population, economy and commissioning of healthcare services is significant (PHE 2019). This rising challenge of MSK conditions has seen it recognised in the Major Conditions Strategy for the first time, alongside conditions such as cancer, mental health and cardiovascular disease (DHSC 2023).

Post‐pandemic elective care recovery, coupled with significant demand on community MSK services, has contributed to long National Health Service (NHS) waiting lists and adds further importance to optimising MSK health (NHSE 2023). Delayed access to support can lead to poorer health outcomes such as chronic pain, reduced quality of life, and the inability to work (Lewis et al. 2018).

These challenges have seen the NHS 10‐year plan highlight the need to shift care from hospital to community settings, with greater focus on prevention (DHSC 2025). Supported self‐management and health behaviour change proposes to address increasing demands and optimise MSK health in local communities (NHSE 2023). The provision of personalised care has demonstrated a positive impact on health outcomes by empowering people through shared decision‐making and enabling self‐management with lower healthcare utilisation (Deeny et al. 2018). This is underpinned by a person‐centred approach and principles of ‘what matters to me’ through meaningful conversations with individuals to listen, understand and involve them in decisions about their health.

Community Appointment Days (CADs) are a relatively new concept to provide more holistic care by bringing together clinical expertise, community partners and the voluntary sector under one roof to support those living with MSK conditions (HERE 2024). Service evaluation data has demonstrated that CADs offer a potential person‐centred approach to aiding self‐management and improving access, whilst preventing the need for multiple appointments (HERE 2024; Alexander et al. 2025).

**FIGURE 1:**
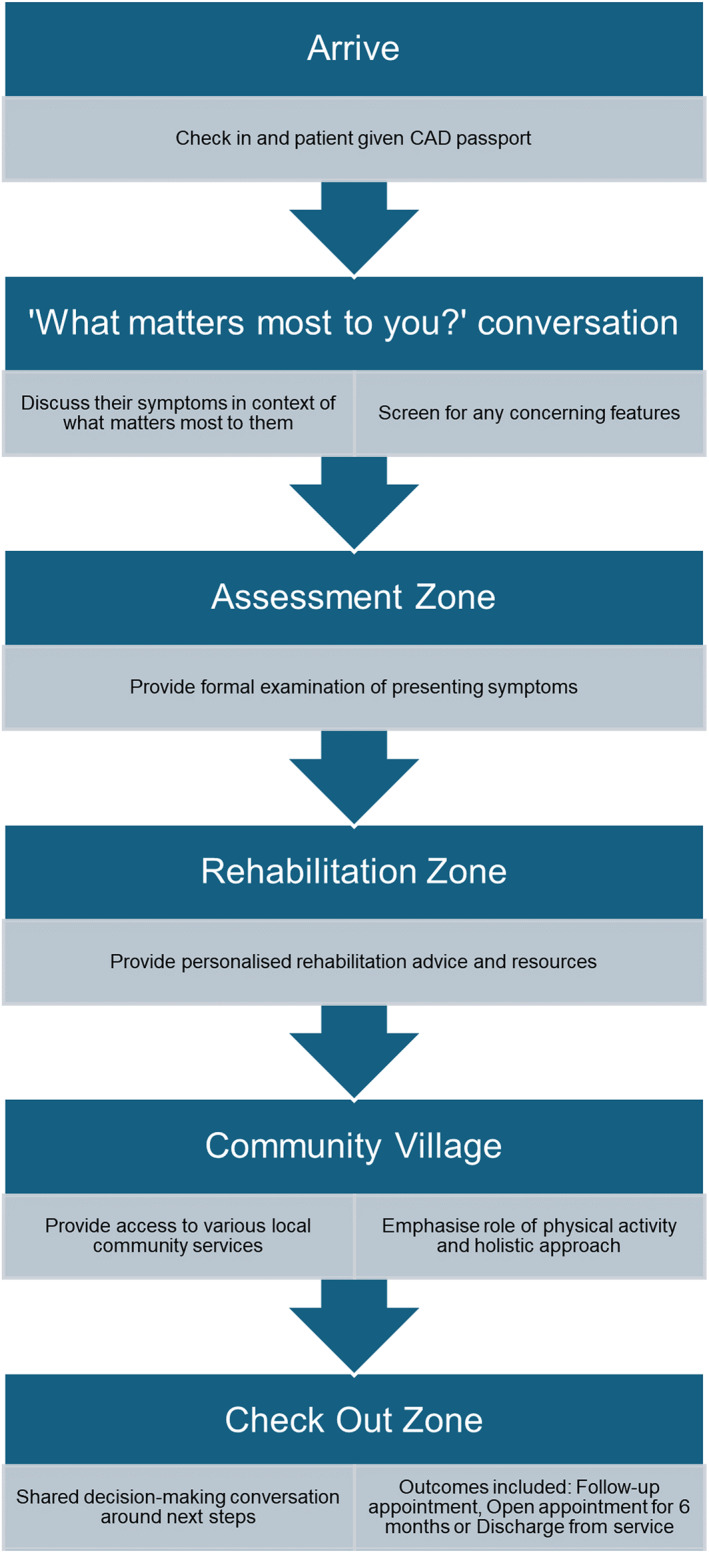
A flowchart of the community appointment day model.

In this evaluation, people on the waiting list for an NHS community MSK service in the Southwest of England were invited to attend their local CAD. The CAD model utilised is shown in Figure 1. Time spent at the CAD was not limited. Patients received a CAD passport enabling documentation of advice received and outcome from the day.

The potential benefits of this new way of delivering MSK care has led to CADs becoming increasingly popular across England, but there remains a paucity of evidence with regards their acceptability and potential impact. The aim of this evaluation was to understand the acceptability of an MSK CAD, by exploring uptake, engagement, outcomes, patient satisfaction and staff experience.

## 2 Methods

### 2.1 Design

The CAD was organised by a local NHS Trust and based in a community centre in the Southwest of England. The evaluation employed a mixed‐methods design.

### 2.2 Ethical Approval

Ethical approval was granted by the Research Ethics Committee at the University of West England (Reference: CHSS.24.06.229) on 11th July 2024. Participant consent was collected by one researcher (AB), prior to data collection.

### 2.3 Sample

Patients were invited to complete quantitative outcome measures before leaving the event. A convenience sample was adopted for the qualitative evaluation, with patients invited via an expression of interest form on the day. Interested patients were contacted by one researcher (AB), via telephone or email. Recruitment continued until the list of interested patients was exhausted. Staff involved in the CAD were invited via email (SI), to attend an online focus group.

### 2.4 Data Collection

Quantitative data including demographics such as age, gender, ethnicity and Index of Multiple Deprivation Code (IMD) were captured. Referral source, duration on NHS waiting list and time spent at the CAD was also collected. Patient experience was measured using the NHS Friends and Family Test. A validated patient‐reported outcome measure called CollaboRATE was utilised to assess level of shared decision‐making (Barr et al. 2014). The greater the score (maximum of 12), the higher level of shared decision‐making experienced.

Experiences of the CAD were captured via patient interviews and a staff focus group. Interview/focus group guides were informed by the theoretical framework of acceptability (TFA) (Sekhon et al. 2017). One researcher (AB) developed the first draft of the interview/focus group guides. These were evaluated by the whole team to ensure questions aligned with the evaluation aim, and TFA domains (Appendix 1 and 2).

One‐to‐one interviews were conducted by telephone or via Microsoft Teams. Three researchers, with previous interview experience (AB, JG, SI), conducted the interviews. The researcher (AB) with most interview experience conducted the first interview, which was reviewed by the remaining interviewers prior to a meeting to discuss the interview approach. Interviews were recorded on encrypted audio recorders or via Microsoft Teams. Patients were unknown to the interviewers.

The focus group was scheduled for 90 min and conducted by two researchers (AB and JG), via Microsoft Teams with cameras turned on.

Interview/focus group recordings were securely transferred to an independent transcription service. Transcripts were reviewed for accuracy and anonymised by members of the research team (JG, IS, SI, EN, SJ), with oversight from another researcher (AB). Data were securely stored, on a password protected, NHS server, only accessible by the study team.

### 2.5 Data Analysis

Descriptive analysis of demographic and outcome data was completed by two researchers (SJ, EN). Qualitative data analysis was undertaken following the six steps described by Braun and Clarke (2013), utilising NVivo (version 14) software. Prior to the commencement of coding, one transcript was coded by four researchers (AB, JG, IS, SI), who then met to review intra‐coding consistency. A hybrid inductive/deductive approach (Fereday and Muir‐Cochrane 2006) was employed. An initial deductive coding of the data was completed considering the TFA domains. The study team then met to reflexively discuss the codes generated. At this stage, an inductive approach was employed, to actively reflect on the codes generated and identify meaningful themes. A consensus was reached on themes and sub‐themes generated. The approach was mirrored by three authors (AB, SJ, EN) during the analysis of focus group data. The two datasets were combined to formulate final codes, sub‐themes and themes.

### 2.6 Reflexivity

The research team consisted of six members, including two (AB, JG) not involved in planning or delivery of the CAD. Of these, AB is employed as an Associate Professor in Rehabilitation at a local University, having no affiliation with the NHS Trust conducting the CAD, and JG is a National Institute of Health and Care Research Pre‐Doctoral Clinical and Practitioner Academic Fellow employed by the host Trust. Planning and delivery of the CAD project was led by SI, whilst SJ, IS, EN were engaged in the CAD as service providers.

## 3 Results

The CAD was held in July 2024. In total, 160 people from the local community MSK waiting list were booked to attend the CAD and 130 (81%) people attended, with 19% (*n* = 30) either cancelling or not attending on the day. Age varied from 17 to 83 years, patients were mostly female (57%), and White British (89%). Further demographic data is shown in Table 1. The Friends and Family test demonstrated that most patients were ‘extremely likely’ (*n* = 63, 65%) or ‘likely’ (*n* = 32, 33%) to recommend a CAD (Table 2). The average CollaboRATE score was 10.8/12 (Table 3).

**TABLE 1:** Demographic data of patients who attended the community appointment day.

*N* = 130	
Age	Range: 17–83 years (mean: 58 years)
Gender	57% female, 43% male
Ethnicity	89% White British 5% Not stated 4% White (other) 1% Asian/Asian British (Indian) 1% Black/Black British (other)
Referral source	61% General practitioner (GP) 20% First contact physiotherapist (FCP) 9% Self‐referral 5% Secondary care 3% Interface service 2% Urgent care
Most common presentations	22% Lumbar spine 12% Cervical spine 20% Knee 15% Hip 14% Shoulder 17% Other
Average time spent at CAD	53 min, range: 15–130 min
Average time waited from referral	175 days, range: 3–223 days
Outcome	Open appointment: 45% Follow up booked: 28% Discharge: 27%
Deprivation index (range 1–10)	Mean: 5 (SD: 2.5)

**TABLE 2:** Friends and Family score.

Would you recommend attending a community appointment day to your friends and family?	
*N* = 97	
	% (*n*)
Extremely likely	65 (63)
Likely	33 (32)
Neither likely nor unlikely	1 (1)
Unlikely	1 (1)
Extremely unlikely	0 (0)
Don't know	0 (0)

**TABLE 3:** CollaboRATE scores following community appointment day.

Question	*N*	Range	Average (SD)
How much effort was made to help you understand your health issues?	97	1–4	3.60 (0.59)
How much effort was made to listen to the things that matter most to you about your health issues?	97	2–4	3.61 (0.51)
How much effort was made to include what matters most to you in choosing what to do next?	97	2–4	3.58 (0.59)
Total average across all three questions			10.8/12

In total, 13 patients were interviewed. Demographic data are presented in Table 4. Interviews took place in September and October 2024. The average length of interview was 22 min (range 16–35 min) (*n* = 9, data not available for four interviews). Two interviews took place via Teams, all others (*n* = 11) took place via telephone. Ten staff members attended the focus group, which took place in September 2024 (see Table 5).

**TABLE 4:** Demographics of patients interviewed.

*N* = 13	
Gender	*n* (**%**)
Male	5 (38.5)
Female	8 (61.5)
Ethnicity	*n* (**%**)
White British	10 (77)
White other	1 (7.7)
Not stated	2 (15.3)
Age	*n* (**%**)
< 40	1 (7.7)
40–49	1 (7.7)
50–59	2 (15.3)
60–69	4 (30.8)
70–79	5 (38.5)
Reporting condition	*n* (**%**)
Lumbar spine	2 (15.3)
Cervical spine	3 (23.1)
Shoulder	3 (23.1)
Ankle	1 (7.7)
Foot	1 (7.7)
Hip	3 (23.1)

**TABLE 5:** Staff involved in the focus group following CAD.

Job title	Zone at CAD
Admin—Team lead	Check in
First contact physiotherapist (FCP)	What matters most
First contact physiotherapist (FCP)	What matters most
MSK^a^ physiotherapist	Assessment
MSK^a^ hub lead	Assessment
MSK^a^ physiotherapist	Rehabilitation
Active partnership lead	Community village
MSK^a^ service lead	Check out
MSK^a^ operational manager	Check out
Community centre manager	n/a

^a^ Musculoskeletal.

Themes and sub‐themes from the qualitative evaluation, and how these aligned to TFA domains, are shown in Table 6 below. Quotations from patients interviewed are presented as ‘P’, followed by their unique identifying number and staff who attended the focus group are presented with an ‘S’.

**TABLE 6:** Overview of themes and sub‐themes, with related TFA constructs.

Theme	Sub‐theme	TFA constructs
Theme 1: A positive response to the ethos of the CAD by patients and staff	a) A great experience of personalised care	AA/IC/PE
	b) Value in having the time to talk	AA/E
	c) A different approach to care	AA/E
	d) Increased visibility of community services	AA/IC
	e) Strengthening knowledge and building confidence to self‐manage	SE/E
Theme 2: The importance of effective planning and communication to ensure the purpose of the CAD model is clear for staff and patients	a) Lack of information and understanding about the day for patients	PE/IC
	b) Importance of staff awareness and understanding of the day	PE/B
	c) Navigating patient expectations and the relevance of triaging processes	E/PE
Theme 3: Effective implementation of the CAD: Practicalities on the day	a) The community setting was valued	AA/OC
	b) A smooth flow to the day was appreciated	AA/PE/B
	c) Access to medical records and information for the patient to take home would have strengthened the experience	AA/B
Theme 4: Potential impact and integration with existing MSK pathways	a) Ability to arrange relevant follow‐up appointments on the day was welcomed	OC/B
	b) Importance of identifying the most appropriate community organisations to invite	PE/IC
	c) Considering the implications for follow‐up capacity, post CAD	OC/B
	d) Mixed opinion on frequency of future CADs	AA/PE

Abbreviation: AA, Affective Attitude; B, Burden; E, Ethicality; IC, Intervention Coherence; OC, Opportunity Costs; PE, Perceived Effectiveness; SE, Self‐efficacy.

### 3.1 Theme 1: A Positive Response to the Ethos of the CAD by Patients and Staff

#### 3.1.1 A Great Experience of Personalised Care

Patients were very positive about their experience of attending the CAD, which they felt improved access to support, and increased awareness of community services available locally. This was seen as a more efficient way to deliver care.

It was a really good day. It was really well thought through, really well organised.

P024

It was bloody amazing. …so well‐orchestrated. Within five minutes of arriving, we understood exactly what it was. And what unfolded thereafter was quite amazing in terms of the number of people that were available. Wow, this is a national health system on steroids!

P102

I think it’s the best idea they’ve ever had.

P057

The ethos of a personalised approach underpinning the CAD was particularly appreciated by patients.

I wasn't left out of anything. I was always talked to, not talked around, if you know what I mean.

P069

All the questions were personal to me… they were all centred around me. My appointments with physios have been a chat, handed a set of exercises and moved on, whereas I felt this time, attention was paid to the detail.

P017

Staff also felt the CAD was an excellent example of holistic patient care and a positive experience that continued beyond the day.

Overall, it was blooming brilliant…. what a wonderful thing to do for patients, how lovely for them to…get all this extra holistic treatment…. it’s fantastic

S6

Positive attitude that really shone through on the day… was quite infectious…. I feel like people are still feeling like it’s positive and there was quite a lot of talk about it amongst other teams

S5

#### 3.1.2 Value in Having the Time to Talk

Participants felt they had ample time during the CAD to understand the information provided and ask questions at their own pace.

You had all the time to speak to them. I never felt like they were trying to move me along for the next person to come up. …there was just no rush whatsoever, which was really nice.

P004

It seemed to be longer and explained better than in the hospital.

P013

Staff also appreciated this relief of time pressure enabling a more patient centred approach to care.

….it was just the time; you just really gave the person that time to sit with them and not have to rush them through figuring out what they really wanted, or they needed

S4

However, the pace of the day was too rushed and overwhelming for some.

It moved on pretty quick from one step to another, I didn't really get a chance to kind of take in everything that was going on around me.

P024

#### 3.1.3 A Different Approach to Care

For staff, there was a strong sense of team unity throughout the day, with First Contact Physiotherapists (FCPs) who normally work alone particularly enjoying a more integrated approach.

To work a bit more as a team …certainly as a group of FCPs rather than being on your own, everyone kind of coming together I thought was really positive

S2

You’re part of something bigger than just yourself in your clinic room…

S7

The change of environment encouraged staff to consider different ways to facilitate supported self‐management.

Made me approach things differently with them, because I wasn’t in the physio setting, I was in the community setting as well, so I was thinking more broadly.

S8

I think the value outweighs the time spent… (planning etc)

S9

However, some staff found the change of working difficult.

it was quite challenging…I wouldn’t say that I maybe had the experience that some others had

S1

Patients described a positive impact of seeing others with similar issues.

I felt that open plan approach is much better, because I think you feel more confident when you know people are going through similar things.

P017

Others highlighted a preference for more privacy.

But to start with, I did just feel a bit, like, anxious about speaking about your health in an open space like that.

P113

#### 3.1.4 Increased Visibility of Community Services

Having access to a range of community services was seen as beneficial by both patients and staff.

There was a lot of that stuff in there I didn’t know existed and it opened my eyes to what other support there is available. Wouldn’t have found out about health coaches… it’s not just being able to see the physio on that day, but it was all the added extras.

P057

By having everyone in the same place, it’s so much easier…and ultimately the patient’s going to get better care as a result

S2

#### 3.1.5 Strengthening Knowledge and Building Confidence to Self‐Manage

Patients described receiving good explanations and information regarding their condition and management, particularly with regards to exercise advice and demonstration.

He explained best way to do it, you know, using a wall. And, you know, he taught me quite a bit, really.

P013

Patients described how attending the CAD gave them hope and confidence to regain control of their health.

So, it’s all come out of this is… is also a… an almost a… a mind changing session… on how to deal with what you’ve got… it’s not just the physical it’s the mental as well.

P057

Staff also felt that the environment promoted self‐management.

it was very clear that most patients were really happy to…self‐manage

S7

It de‐medicalised things… the whole day was catered towards promoting that self‐empowerment, and I think the community village is really important for that too

S4

### 3.2 Theme 2: The Importance of Effective Planning and Communication to Ensure the Purpose of the CAD Model Is Clear for Staff and Patients

#### 3.2.1 Lack of Information and Understanding About the Day for Patients

Some patients didn't know what to expect prior to attending the day. They felt the invitation letter lacked clarity and detail about the nature of the day and purpose of the invite.

It just wasn’t… didn’t come over in the letter that I received that that was what was happening… I just totally misunderstood what I was going for really.

P108

I’d managed to get onto this, and I don’t know why I got the invitation above other people

P016

Patients would have liked more detailed information beforehand.

I thought it was an actual appointment at three o'clock to see a physio. I wasn't sure, I don't know whether I had missed some information or what… just warning people that they've got all those services to go to, so they allow more time

P113

#### 3.2.2 Importance of Staff Awareness and Understanding of the Day

Staff reflected that communication to the team regarding the purpose of the CAD prior to the event could have strengthened understanding.

Although it has the benefit of tackling the waiting list, that wasn’t ever the actual aim…it was to get people to the right place straight away. …I guess it’s how we communicate that … and make sure that everybody is very aware of that, rather than it feeling like something else before we start

S7

Staff would have liked to be more involved in the planning process to enable appropriate training, align expectations and improve efficiency on the day.

From an admin perspective…it didn’t give us enough time to actually plan it as well as I would have liked it to

S1

There was agreement that a briefing on the morning of the CAD would have been appreciated to enable familiarisation.

I don’t work for the NHS…so I think a debrief [sic] on the actual morning to introduce people to each other would have been a good idea

S6

I don’t think we really had a feel for each other’s roles, we were kind of there, one cog in that machine, so it would be nice to maybe spend a bit of time in those different sections as well

S4

#### 3.2.3 Navigating Patient Expectations and the Relevance of Triaging Processes

Patient expectations were not always met by attending the day. Some wanted more thorough investigations and detailed explanations to better understand the root cause of their symptoms.

I need to see like proper doctor as well, to look at it like, to do a proper x‐ray and stuff like that. But it was just exercise. That's it. Nothing else she could do about it… maybe a bit more detail would have been good, or a bit more assessment.

P058

Staff highlighted that some patients were less likely to benefit from the day due to needing more support than the CAD could provide.

There were people there that they had lots of other medical things going on and probably not appropriate for that environment on the day, and I think it was managed really well by the clinicians, but too many of those could probably steer it in the wrong direction, but that is more planning.

S4

Some staff felt there needed to be a more accurate triage process to ensure only appropriate patients were invited. Others didn't feel it was necessary to restrict access to the CAD.

Trying to figure out how we’d filter out the most appropriate people to come to that type of day

S4

Straightforward referrals might be better suited, but actually …. it’s often the more complex patients who require access to multiple services

S5

### 3.3 Theme 3: Effective Implementation of the CAD: Practicalities on the Day

#### 3.3.1 The Community Setting Was Valued

Providing services in the community rather than a hospital was regarded as positive by both patients and staff.

You go to a hospital, and you always think God, I’m ill, but you’re not ill, you’re going to see somebody about a set of exercises to improve a condition that you’ve got… I just felt the whole setup was much better.

P017

…keep it out of our hospital as humanly possible

S7

#### 3.3.2 A Smooth Flow to the Day Was Appreciated

The flow of the CAD was key to ensuring that people understood the purpose of each zone and were able to access the range of services based on what mattered most to them.

I thought it was fantastically organised. From one section to another, it was great.

P069

Handovers between staff and use of signage to direct patients between different zones enhanced the experience.

They didn't say go there, go there. Each person took you to the relevant part.

P004

Staff agreed on the importance of appropriate placing of skill mix in each zone and use of experienced clinicians at the start of the patient journey.

I think it worked really well having the Band 7s and FCPs at the start of the journey and then kind of filtering it down, because they’re seeing the people that have got most experience in the beginning

S4

However, some staff felt that separation of the zones was a potential barrier that created repetition of information and suggested making the process more streamlined.

Potentially linking the initial consult conversation with the assessment might help streamline things, …they would come speak to us, and then they’d go to the assessment team and repeat quite a lot of the same questioning and information

S2

#### 3.3.3 Access to Medical Records and Information for the Patient to Take Home Would Have Strengthened the Experience

It was considered important that staff had access to patient health records, including any relevant investigations. Some patients were unsure if records were available on the day.

I think there was some record of me there. How much, I’m not really sure.

P016

Staff agreed that access to electronic patient records would have been beneficial for history taking, onward management and reduction of tasks to complete beyond the day.

If we had access to EMIS [electronic patient record]… that would help you tailor things to them a bit more without relying on them having to remember everything

S4

Access to imaging or orthopaedic interface referral would…streamline care…quite a few of us ended up having a list of patients to take away to… get stuff sorted

S2

A patient passport to record relevant personalised information to support self‐management was provided and sent to patients retrospectively. Patients described how they would have preferred to have paper copies to take away with them on the day.

Whilst they told me at the time what they said, I mean, I didn’t get them for about three weeks. Three weeks later, I’m not going to remember every word.

P057

### 3.4 Theme 4: Potential Impact and Integration With Existing MSK Pathways

#### 3.4.1 Ability to Arrange Relevant Follow‐Up Appointments on the Day Was Welcomed

Patients felt the exercise prescription from CAD helped whilst awaiting any follow‐up, demonstrating integration between the CAD and existing MSK pathways.

I had the exercises here that I could start so that when I went to the actual physio, I could point out the ones that I found easy to do, that I could do… the ones I couldn't do, I could relay to them the reason why it wasn't working for me

P004

A further demonstration of streamlined care was given with one patient who was directly referred into secondary care for a total hip replacement.

It was identified what the issue was, and then it was referred on for hip replacement, which was all arranged. And couple of weeks’ time, I’ll be going in for it.

P069

Patients appreciated the ability to be referred into community exercise referral schemes, physiotherapy, secondary care services, or back into primary care where appropriate.

At the end they made the appointment with you to have the ongoing treatment. It wasn’t just, this is the day and that’s it

P057

Some patients had difficulty arranging a follow‐up due to online issues or failing to make contact prior to the deadline.

I tried doing it and it wouldn't actually allow me to do it, and I needed to ring so I actually missed that deadline. I thought I had done it online, but I didn't, so that part of it wasn't great.

P113

#### 3.4.2 Importance of Identifying the Most Appropriate Community Organisations to Invite

Utilisation of some services within the community village was low, and the importance of inviting the most relevant organisations was highlighted.

With hindsight some of them were probably inappropriate to invite, I think the ones that got on better were the ones that were particularly involved in patient care and had more to do with health

S6

#### 3.4.3 Considering the Implications for Follow‐Up Capacity Post CAD

A minority of patients were unsure about what would happen following the CAD, with some patients wanting a follow‐up but being unsure how to arrange this.

Don’t know how to make an appointment, be honest with you. Because now I have to… I have to go hospital or I have to go ring my GP, or whether… there's no direct number

P058

A follow‐up letter was suggested to check up on patients following the CAD.

Maybe a letter to remind you of what you have to do sent through the post… just to say if you need to see anybody… just have the details on.

P113

#### 3.4.4 Mixed Opinion on Frequency of Future CADs

Patients liked the idea of being able to access future CADs if needed.

If that could be integrated, I don’t know, quarterly, half‐yearly, something like that, I think it would help people a lot.

P017

Staff also felt that repeating the CAD would be a good idea.

I think every three to four months would probably be realistic

S9

if we’ve got our four hubs and we’re thinking do we run a CAD from each hub and we get set up with a regular location that’s ready to go… then we could do it more frequently

S5

However, there was concern that the experience of the day would change if it was done more frequently and could potentially place added pressure on staffing levels.

If doing these events regularly and it’s not new and exciting… some of the enjoyment would be lost, certainly if we’re scaling numbers and we’re refining staffing and there are more pressures…

S10

## 4 Discussion

This evaluation demonstrated a positive experience from patients and staff in relation to the ethos of the community appointment day (CAD) model. The personalised and holistic approach was particularly valued, alongside the visibility of community services and potential to support self‐management.

Patients felt listened to, had time to talk, and were involved in decisions about their condition. The patient reported CollaboRATE measure (Table 3), demonstrated excellent shared decision‐making (SDM) and is consistent with other CAD service evaluation data from Sussex (HERE 2024). A systematic review found that physiotherapists can inadvertently adopt a paternalistic approach at times, which can negatively impact SDM and health outcomes (Grenfell and Soundy 2022). A shift from ‘consultation to conversation’ aims to focus on what matters most to the individual and may help foster self‐management. Adopting this personalised approach may require additional training for staff and this should be considered during the planning of a CAD. Time is often cited as a barrier to delivering SDM (Thompson et al. 2024). A key strength of the CAD model is its flexible appointment duration, with the average time spent at the CAD (53 min) longer than most NHS MSK consultations. This impact of affording time should not be underestimated, being acknowledged by both patients and staff as a key benefit of the CAD.

It is important that CADs are planned appropriately with relevant stakeholders engaged from the start. Building relationships with local Active Partnerships may aid awareness of local community services and help support self‐management through promoting physical activity (Davies et al. 2019). Identifying optimal community partners should be aligned with local health needs and consideration of the impact of MSK conditions on broader health determinants, such as physical activity, mental health, employment support, and weight management. Having access to this support appeared to boost patients' knowledge and confidence to self‐manage. The importance of effective communication with regards the format of the CAD model warrants consideration as some patients did not know what to expect and improving this may help enhance attendance rates. Suggestions for future CADs include making it clear how it differs from a typical MSK appointments, and provision of details about the set‐up of the day, including the anticipated length of time for the visit.

The integration of the CAD model into existing MSK pathways is critical if it is to add value to patients, staff, and the commissioning of MSK services. Just over a quarter of those attending the CAD (28%) required a follow‐up in the local MSK service, indicating its potential value in supporting self‐management. Being able to arrange necessary follow‐up appointments on the day or referrals to appropriate services, such as orthopaedics, added to the positive experience. There was no consensus on the optimal frequency of delivering CADs, but a balance is required regarding their potential impact on improving waiting times and the capacity of the community and voluntary sector to support their delivery. Further research into longer term impact on health outcomes and healthcare utilisation of those attending CADs is recommended.

Those invited to the CAD in this service evaluation were not triaged with regards their suitability. Stratification tools are often utilised in MSK services to identify those at risk of chronicity or a poor outcome (Hill et al. 2020). These individuals might be considered inappropriate for the CAD given the environment, but it could be argued that having earlier access to a wide range of services co‐located through the CAD model may provide a more holistic approach to those with more complex MSK health needs.

**FIGURE 2:**
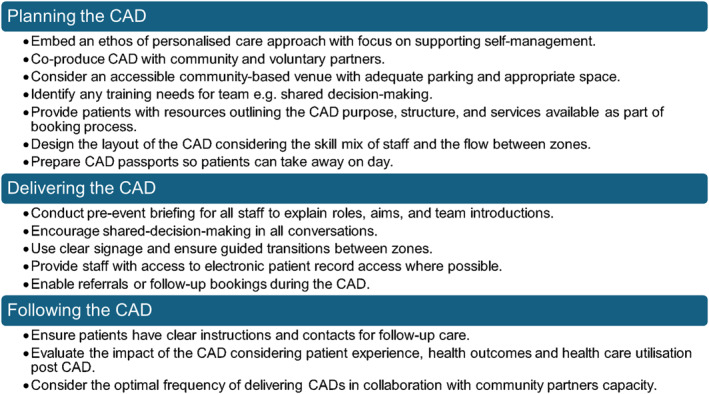
Considerations for delivering community appointment days.

Due to the increasing demand and complexity seen across healthcare, there is growing emphasis on collaboration between NHS services and community‐based organisations. The CAD model is designed with this ethos in mind as it brings services together under one roof. It can also improve access and help reduce waiting lists which aligns with the National Health Service England (NHSE) Improvement Framework for community MSK (NHSE 2023). Access to advice, assessment and rehabilitation alongside other services such as talking therapies, health checks, and local leisure providers was appreciated by the patients in this evaluation. The improved visibility of local community services was highlighted and this cross sector working provides an example of how future healthcare delivery can meet government plans to shift care from hospital to a community setting (DHSC 2025). A summary of considerations when planning to deliver a CAD is shown in Figure 2.

## 5 Limitations

It is acknowledged that findings might not reflect those of the wider population of patients with MSK conditions. There was a lack of ethnic diversity amongst patients attending, and this could also impact of the transferability of findings. While the findings indicate that the CAD may support self‐management, a longer follow‐up period of data collection, particularly considering re‐referral routes and frequency of follow‐up appointments, would be beneficial.

## 6 Conclusion

The CAD model was widely accepted by patients and staff as an effective, personalised and holistic model of care. Key strengths included having time to meaningfully engage, an emphasis on shared decision‐making, and the enhanced access to community and voluntary services. These factors contributed to increased patient confidence to self‐manage and reduced need for follow‐up appointments. Staff appreciated the positive team approach and opportunity to deliver care in a collaborative, community‐based setting. Successful implementation requires effective planning, stakeholder involvement and improving patient awareness of the CAD model. Future research should explore long‐term outcomes of the CAD model on healthcare utilisation, and its suitability for those with more complex needs. Additionally, establishing optimal frequency, integration within MSK pathways, and the potential role of stratification tools will be fundamental for refining and scaling the model sustainably.

## Author Contributions

SI and AB devised the study design. AB, JG, SI carried out data collection. Data analysis and interpretation was conducted by JG, IS, SI, EN, SJ with support from AB. All authors contributed to the writing and review of the manuscript.

## Conflicts of Interest

The authors declare no conflicts of interest.

## Data Availability

Research data are not shared.

## Appendix 1

### How Acceptable Is a Community Appointment Day (CAD) for People With Musculoskeletal Conditions

**Intro:** Thank you very much for your time today…

**Aim of session**

- Understand your experiences of the CAD,
- Hear your thoughts and views on the day
- Confidential—whole session will be kept completely confidential and anonymous. This session will be transcribed, all identifiable data removed, ID numbers assigned, and then the recording will be deleted.

Any questions?

**Table:**

	Interview topic guide
(Attitude)	How do you feel about the day, now that you've attended it.
(Attitude)	How do you feel about attending another day in the future?
(Self‐efficacy)	How confident are you that you can follow the advice and suggestions you received on the day?
(Likes/dislikes)	What did you like/dislike?
(Intervention coherence)	**Was it clear?**—Do you understand the discussion and advice you received, and what each area was trying to help you with?
(Ethicality)	The aims of the CAD included—being able to get an appt sooner, and being able to spend more time talking about your needs and getting support/advice—were these aims meaningful to you?
	Do you value these things—(knowledge/skills/setting goals/social support)
(Perceived effectiveness)	Do you think the CAD will help you to self‐manage your condition?
	What about over the longer term?
(Burden/intended use)	How do you feel about the amount of time and effort that was required to attend the day?
	Do you think you will use all the advice and connections?
	What would stop you from attending a CAD in the future?
(Opportunity costs)	Do you think the potential benefits of attending the day, outweigh the amount of time and effort needed to attend? Was it worth it?

**Closing**: Thank you very much for taking part, do you have any other reflections about anything we have discussed, or any other questions?

## Appendix 2

### How Acceptable Is a Community Appointment Day (CAD) for Staff

#### Focus Group Guide

Thank you very much for taking the time to attend today

**Aim of session**

- Understand your experiences of the CAD,
- Identify Barriers/facilitators impacting future CAD events (acceptability, service sustainability, implementation)

**Ground rules**

- Please use hand up function on teams—please allow others to speak. We hope to hear a range of views.
- Introduce—name/place of work—in chat.
- Confidential—whole session will be kept completely confidential and anonymous. This session will be transcribed, all identifiable data removed, ID numbers assigned to each voice, and then the recording will be deleted.

Approach of today's session—wider/bigger picture than the earlier feedback session held after the CAD. More about thoughts/attitudes/opinions/view, rather than a focus on practical changes.

**Table:**

(Attitude)	How did you **feel** about taking part in the CAD, and how do you feel about taking part in another one in the future?
(Self‐efficacy)	How **confident** were you in your role for the day—was it clear?
	Any need for additional support/training/clarification prior to future CADs?
(Likes/dislikes)	What did you **like/dislike?**
(Intervention coherence)	Did you **understand the concept** of the CAD, was the **purpose of each section clear and relevant** from your perspective?
	Would you make any changes to the day to make it more straightforward for staff?
(Ethicality)	Do you think **the aims** of the CAD (i.e. patients being able to get an appt sooner, being able to spend more time talking about their needs and getting support/advice) are **relevant to your patient population**.
	Any suggestions for **running it differently** in the future?
(Perceived effectiveness)	Do you think the CAD will help patients to self‐manage their condition? What about over the longer term? Anything else needed (outside of the CAD?)
	How should we measure if it's effective? What is most important?
(Burden/intended use)	How do you feel about the amount of **time and effort** that was required to attend the day?
	What would stop you from taking part in CADs in the future?
	Has anybody experienced any potentially unintended consequences (service or patient perspective?)
(Opportunity costs)	Do you think the **potential benefits** of taking part in the day, **outweigh the amount of time and effort** needed to attend?
	Was it worth it?
	Should we explore repeating this/how often?
Sustainability/implementation	What do we need to consider for ongoing events.
	How do we keep it going?

**Any other thoughts/suggestions/reflections** about anything we have discussed, or any other questions?

**Closing**: Thank you very much for taking part.
